# Serum IL-6, IL-10, TNF-a changes in postpartum perineal oedema rehabilitation

**DOI:** 10.5937/jomb0-56599

**Published:** 2025-07-04

**Authors:** Lili Xue, Yaqiong Jiang, Yongli Guo, Hongyan Ma, Lingling Jiang, Xiaoyu Wang, Xinyi Kang, Ying Wang, Jiachen Cao, Liping Chen

**Affiliations:** 1 Affiliated Hospital 2 of Nantong University, Department of Obstetrics and Gynecology, Nantong First People's Hospital, Nantong, Jiangsu, China

**Keywords:** CRP, ESR, IL-6, IL-10, TNF-a, natural childbirth, Perineal oedema, manual lymphatic drainage, abdominal breathing, postpartum recovery, CRP, ESR, IL-6, IL-10, TNF-a, prirodni porođaj, perinealni edem, manuelna limfna drenaža, abdominalno disanje, postpartalni oporavak

## Abstract

**Background:**

This study aims to evaluate the effectiveness of manual lymphatic drainage (MLD) combined with abdominal breathing in rehabilitating postpartum perineal oedema and Serum IL-6, IL-10, TNF-a.

**Methods:**

A total of 172 primiparous women who delivered in our hospital between January 2022 and June 2023 were randomly assigned to either the observation group (n=86) or the control group (n=86). The control group received routine midwifery care, while the observation group received additional MLD and abdominal breathing training. Outcomes measured included pain levels, induration diameter, comfort, emotional state, recovery time, and clinical efficacy. Inflammatory markers, including C-reactive protein (CRP), erythrocyte sedimentation rate (ESR), interleukin-6 (IL-6), interleukin-10 (IL-10), and tumour necrosis factor-alpha (TNF-a), were also assessed.

**Results:**

Both groups demonstrated significant reductions in Self-Rating Anxiety Scale (SAS) and Self-Rating Depression Scale (SDS) scores after treatment (P<0.05), with the observation group showing a greater decrease (P<0.05). The observation group exhibited a significantly shorter recovery time (3.6±1.8 days vs. 4.2±2.5 days, P<0.05) and reported higher comfort levels and lower pain scores than the control group (P<0.05). The effective treatment rate was 97.68% in the observation group, significantly higher than the 82.56% observed in the control group (P<0.05). Inflammatory markers, including IL-6 and TNF-a, showed a significant reduction in the observation group.

**Conclusions:**

Combining MLD and abdominal breathing reduces postpartum perineal oedema effectively, alleviates pain, and enhances recovery. It also lowers inflammatory markers (IL-6, TNF-a) and increases IL-10, promoting faster healing and improved maternal comfort.

## Abstract (sr)

**Uvod:**

Cilj ove studije je da se proceni efikasnost manuelne limfne drenaže (MLD) u kombinaciji sa abdominalnim disanjem u rehabilitaciji postpartalnog perinealnog edema i serumskih vrednosti IL-6, IL-10, TNF-a.

**Metode:**

Ukupno 172 žene koje su se prvi put porodile u našoj bolnici izme|u januara 2022. i juna 2023. godine, nasumično su raspore|ene u grupu posmatranja (n=86) ili kontrolnu grupu (n=86). Kontrolna grupa je primala rutinsku negu babica, dok je grupa koju smo posmatrali imala dodatnu obuku iz MLD-a i abdominalnog disanja. Mereni su rezultati kao što su nivo bola, prečnik induracije, udobnost, emocionalno stanje, vreme oporavka i klinička efikasnost. Takođe su procenjeni upalni markeri, uključujući Creaktivni protein (CRP), brzinu sedimentacije eritrocita (ESR), interleukin-6 (IL-6), interleukin-10 (IL-10) i faktor nekroze tumora-alfa (TNF-a).

**Rezultati:**

Obe grupe su pokazale značajno smanjenje skorova na Skali samoprocene anksioznosti (SAS) i Skali samoprocene depresije (SDS) nakon tretmana (P<0,05), pri čemu je grupa posmatranja imala veći pad (P<0,05). Grupa posmatranja je imala značajno kraće vreme oporavka (3,6±1,8 dana naspram. 4,2±2,5 dana, P<0,05) i prijavila veći nivo udobnosti i niže skorove bola u poređenju sa kontrolnom grupom (P<0,05). Efikasnost tretmana bila je 97,68% u grupi koja je posmatrana, značajno viša nego u kontrolnoj grupi (82,56%) (P<0,05). Upalni markeri, uključujući IL-6 i TNF-a, su pokazali značajno smanjenje u grupi koja je posmatrana u odnosu na kontrolnu grupu (P<0,01), što sugeriše pozitivan uticaj na rešavanje upale i zaceljivanje tkiva.

**Zaključak:**

Kombinacija MLD-a i abdominalnog disanja efikasno smanjuje postpartalni perinealni edem, ublažava bol i pobolj{ava oporavak. Takođe smanjuje upalne markere (IL-6, TNF-a) i povećava IL-10, što podstiče brže zaceljivanje i poboljšava komfor majke.

## Introduction

Childbirth, particularly vaginal delivery, is often considered the most natural and physiological way to bring a fetus into the world. However, it is also associated with a range of potential complications, especially when the mother’s condition is unfavourable. One of the most common and distressing postpartum complications is perineal oedema, which can result from local trauma, such as episiotomy or tearing during delivery. This condition is frequently observed in the first few days postpartum and is a major cause of discomfort and delayed recovery for new mothers. While mild cases of perineal oedema tend to resolve spontaneously within a few days, more severe cases can lead to prolonged swelling, pain, impaired circulation, skin thinning, and an increased risk of infection, all of which significantly hinder postpartum recovery [1] [2] [3] [4]. Therefore, effective treatment strategies for perineal oedema support maternal recovery and improve the quality of life in the immediate postpartum period [5] [6] [7].

Postpartum inflammation, particularly in perineal oedema, is central to the healing process. During this period, the body is engaged in resolving tissue damage and restoring normal physiological function, but if inflammation becomes excessive or prolonged, it can impede recovery and exacerbate discomfort. Biomarkers such as C-reactive protein (CRP) and erythrocyte sedimentation rate (ESR) are commonly used to assess systemic inflammation [8] [9]. Additionally, cytokines like interleukin-6 (IL-6), interleukin-10 (IL-10), and tumour necrosis factor-alpha (TNF-α) provide more specific insight into the local inflammatory response and immune modulation. IL-6 and TNF-α are pro-inflammatory cytokines that contribute to tissue damage and swelling, while IL-10 has anti-inflammatory properties, helping to balance the immune response and promote tissue repair [9]. Elevated levels of these markers in the postpartum period can indicate ongoing inflammation, which may hinder both tissue healing and maternal comfort. Therefore, understanding how interventions influence these markers is crucial for assessing their effectiveness in reducing perineal oedema and promoting recovery [10].

Manual lymphatic drainage (MLD), a therapeutic technique commonly used to treat lymphoedema, has recently gained attention as a potential treatment for postpartum perineal oedema. MLD uses specific techniques to stimulate lymphatic flow, increase lymph node reabsorption, and reduce interstitial fluid buildup, thereby alleviating swelling. Several studies have demonstrated the effectiveness of MLD in reducing oedema after surgery, particularly in the treatment of vulvar lymphoedema following cancer surgery [11] [12]. Building on this, our study aims to explore the combined effect of MLD and abdominal breathing exercises in rehabilitating postpartum perineal oedema. By targeting both the physical swelling and the underlying inflammatory processes, this combined approach could offer a promising method for improving maternal recovery after vaginal delivery.

This study evaluates the effects of MLD and abdominal breathing on inflammatory markers (IL-6, IL-10, TNF-α) and clinical outcomes in women suffering from postpartum perineal oedema. Our hypothesis is that this intervention reduces local swelling and modulates inflammatory responses, leading to faster recovery and improved maternal well-being.

## Materials and methods

### General clinical data

A total of 172 cases of primipara who gave birth in the delivery room of our hospital from January 2022 to June 2023 were chosen to be the study objects and randomly divided into the observation group and the control group, with 86 cases in each group. Primipara in the control group aged from 22 to 34 years old, with an average age of (26.4±2.1) years. The fetal double parietal diameter was 87–94 cm, with an average diameter of (92.1±2.3) cm. The average gestational age was (38.7±1.2) weeks, ranging from 37 to 41 weeks. Primipara in the observation group aged from 22 to 35 years, with an average age of (27.0±2.2) years. The fetal double parietal diameter was 89=95 cm, with an average diameter of (93.3±2.1) cm. The average gestational age was (38.6±1.3) weeks, ranging from 37 to 43 weeks. No significant difference was discovered in the basic data between 2 groups (P>0.05), which was comparable.

Inclusion criteria: (1) Single fetus, gestational age 37 weeks; (2) Primipara; (3) The fetus was in the head position, and the fetal heart rate was well monitored; (4) Met the indications of vaginal trial birth; (5) Normal communication and communication skills; (6) Informed consent to this study.

Exclusion criteria: (1) Multiple pregnancy; (2) The fetal position was not head position; (3) Predicted fetal body weight 4 kg or double parietal diameter 10 cm; (4) Gestational age<37 weeks; (5) Patients with severe pregnancy complications or who are not suitable for vaginal delivery; (6) Commu ni cation difficulties; (7) Could not cooperate to complete the researcher; (8) 8 halfway to cesarean section; (9) Quit research for various reasons.

### Methods

The control group received routine midwifery nursing. Lithotomy of the bladder was taken for parturient women. When the perineum was tense, the midwife used her right hand to support the puerpera’s perineum inward and upward, and at the same time, used her left hand to assist the delivery of the fetal head. When the contraction was too strong, the midwife guided the puerpera to breathe deeply to reduce intra-abdominal pressure.

The observation group adopted abdominal breathing and MLD techniques based on routine midwifery nursing: (1) Abdominal breathing: The midwife guided puerpera to perform abdominal breathing training during the later stages of pregnancy. Pregnant women took a flat position, relaxed the whole body, deeply inhaled and slowly exhaled deeply, breathing 7 times/min for 5 minutes. When the uterine opening was 2 to 8 cm or every 2 to 4 minutes, the puerpera was instructed to use rapid breathing, relax the whole body, inhale through the nose, exhale through the mouth, and adjust according to the strength of the contraction and the frequency of the breathing rhythm. If the contraction was strong, the breathing rate was increased accordingly; if the contraction was slow, the breathing rate was slowed down. If the contraction became stronger, the breathing rate was also increased accordingly. If the contraction of the uterus was slow, the breathing rate was also slowed down. When the puerpera’s cervix dilated to 8=10 cm or contractions occurred every 90 seconds, the puerpera was instructed to perform shallow breathing training. The puerpera breathed through her mouth, making a »xi« sound in her throat, adjusted her breathing rate according to the intensity of the contractions, inhaled and exhaled four times quickly, and then breathed deeply until the contractions stopped. When the uterine opening was fully opened, the puerpera was instructed to hold her breath, lift her head and chin, inhale deeply, hold her breath for 20 seconds, inhale deeply again, and repeat the above breathing actions until the fetus was delivered. In the labour process, if the force did not reach the standard, the puerpera had no conscious force; the puerpera was guided to relax the whole body, breathe quickly with the mouth, and avoid ineffective force.

(2) MLD: The operator used rotation, stroking and circling methods to drain the labia minor to the pubic mound [1], the pubic mound was drained to the abdominal lymph node area, and the left and right labia major was drained from the bottom to the ipsilateral abdomen, groin or contralateral groin. The technique was light, slow, soft and slow, and the pressure was suitable for the puerpera to bear and avoid touching the perineal wound. The time of each artificial drainage was about 15–20 minutes. The degree of perineal oedema, pain score and induration size were re-evaluated half an hour after drainage. The midwife determined whether additional interventions were needed based on the assessment results.

### Observation indicators

(1) Clinical efficacy evaluation. Evaluation criteria for curative effect: Obviously effective: After the completion of treatment, perineal oedema and other related symptoms improved significantly, the remission range of oedema was more than 80%, and no discomfort symptoms and infection appeared. Effective: After the completion of the treatment operation, the related symptoms of perineal oedema were improved, the regression range of oedema was 25%~80%, and the self-consciousness symptoms were significantly reduced. Ineffective: After the completion of treatment, perineal oedema and other related clinical symptoms were not improved, and the degree of oedema regression was less than 25% or even aggravated.

(2) Evaluation of pain degree of puerpera. The pain degree was evaluated utilising the visual simulation scoring method [4]. In the specific operation process, a scale with a length of 10 cm was prepared, and the maternal was informed that the position of 0 cm meant no pain, the position of 10 cm meant relatively severe pain, and every 1 cm represented 1 point. The puerpera was asked to mark the position on the scale that best reflects her pain degree with a pen. The higher the pain score, the more severe the maternal pain.

(3) Induration diameter evaluation. The induration size of the perineal oedema pain point was measured with a straight ruler.

(4) Comfort evaluation. At the end of the treatment, the responsible nurse conducted a survey on each case’s comfort, divided into four levels: very comfortable, relatively comfortable, generally comfortable and uncomfortable.

(5) Evaluation of maternal emotional status. The self-rating scales for anxiety and depression (SAS and SDS) were implemented to evaluate the negative emotions of 2 groups, in which SAS score above 50 was anxiety, SDS score above 53 was depression.

(6) Evaluation of recovery time and infection of patients in 2 groups. The recovery time and infection of 2 groups were statistically analysed.

(7) Inflammatory marker evaluation.

Blood samples were collected from all participants before and after the intervention to measure inflammatory biomarkers, including C-reactive protein (CRP), erythrocyte sedimentation rate (ESR), interleukin-6 (IL-6), interleukin-10 (IL-10), and tumour necrosis factor-alpha (TNF-α). CRP and ESR were assessed as general indicators of systemic inflammation, while IL-6, IL-10, and TNF-α levels were used to evaluate local inflammatory responses and immune modulation. Changes in these markers were compared between the two groups.

Venous blood samples were collected from all participants at two time points: before the intervention (within 6 hours postpartum) and 48 hours after treatment. CRP, ESR, IL-6, IL-10, and TNF-α levels were measured using enzyme-linked immunosorbent assay (ELISA) and automated haematology analysers. A reduction in CRP, ESR, and IL-6, TNF-α, along with an increase in IL-10, was considered indicative of improved postpartum recovery. The differences between the observation and control groups were analysed to determine the impact of MLD and abdominal breathing on inflammatory resolution and tissue healing.

### Statistical analysis

SPSS 20.0 software was adopted for statistical analysis. The measurement data conforming to normal distribution were exhibited by means of standard deviation. Two independent samples, t-test and repeated measurement ANOVA, were adopted for comparison between groups. The count data was expressed as several cases (%), and the χ^2^ or rank sum test was adopted to compare groups. P<0.05 was considered to be statistically significant.

## Results

### Negative emotion scores between 2 groups

**Figure 1:**
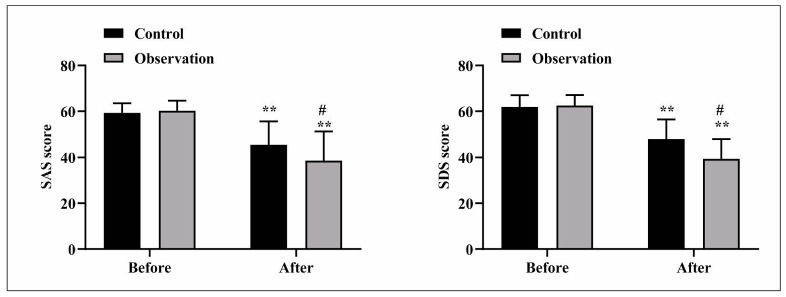
Negative emotion scores between 2 groups. Compared with before nursing, ** was P<0.01; compared with the control group, # was P<0.05.

The scores of SAS and SDS in both groups after nursing presented lower than those before nursing (P<0.05). After nursing, relative to the control group, SAS and SDS scores in the observation group were significantly reduced (P<0.05), as seen in Figure 1.

### Recovery time and infection status of 2 groups

**Figure 2:**
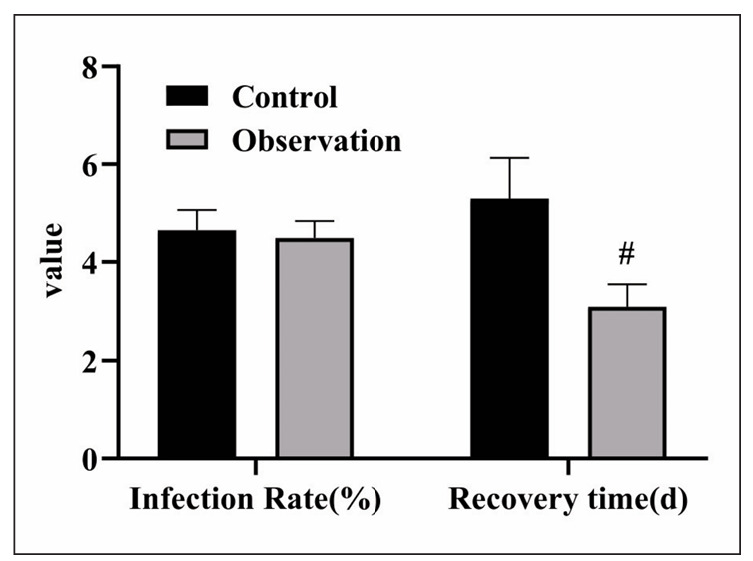
Recovery time and infection status of the two groups. Compared with the control group, # was P<0.05.

The recovery time of the observation group was (3.6±1.8) d, which was significantly lower relative to the control group (4.2±2.5) d (P<0.05). There was no statistical difference in the infection rate between 2 groups (P>0.05), as displayed in Figure 2.

### Comfort level between 2 groups

**Figure 3:**
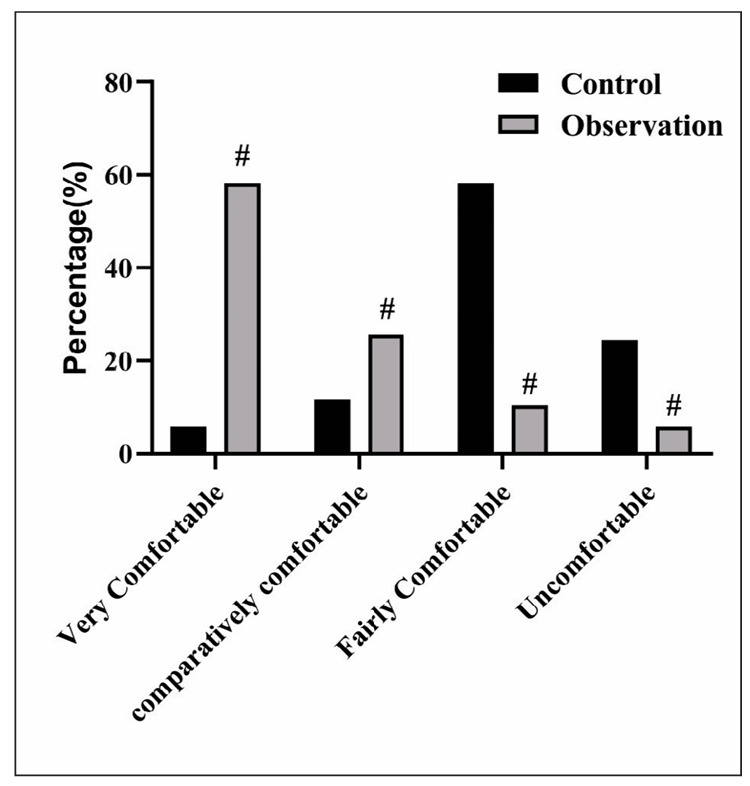
Comfort level between 2 groups. Compared with control group, #was P<0.05.

The comfort level of the observation group was better relative to the control group, with statistical significance (P<0.05), as exhibited in Figure 3.

### Pain degree of puerpera in 2 groups

No significant difference was discovered in the degree of pain before treatment between 2 groups. The degree of pain of parturients in the observation group was slighter after treatment, and there was a statistically significant difference between parturients in the control group and those in the observation group (P<0.05), as displayed in Table 1.

**Table 1:** General data analysis.

Groups	N	Before nursing	After nursing
Observation<br>group	86	5.48±0.87	2.04±0.22
Control group	86	5.39±0.90	3.38±0.35
t		1.415	3.832
P		0.108	0.043

### Comparison of relevant time indexes

**Figure 4:**
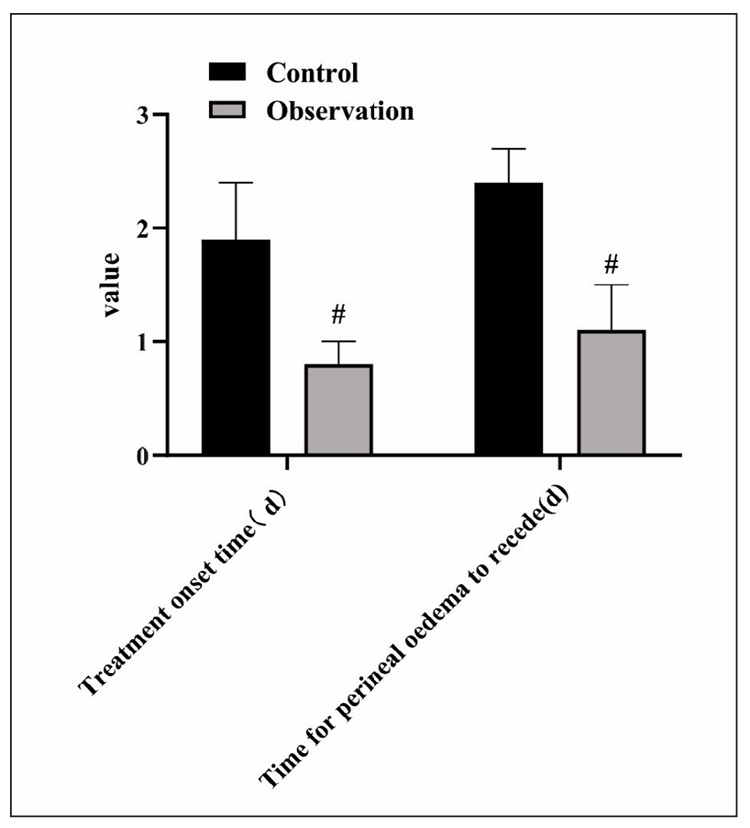
Comparison of relevant time indexes. Compared with the control group, #was P<0.05.

As displayed, the time of treatment onset and perineal oedema regression in the observation group were relatively shortened with the control group (P<0.05). Figure 4

### Evaluation of clinical curative effect

The results showed that the effective rate of treatment in the observation group was 97.68%, presented higher than that of 82.56% in the control group (P<0.05), as exhibited in Table 2.

**Table 2:** Evaluation of clinical curative effect.

Groups	Obviously<br>effective (%)	Effective<br>(%)	Ineffective<br>(%)	Effective<br>rate (%)
Observation<br>group	53 (61.63)	31 (36.05)	2 (2.32)	97.68
Control<br>group	38 (44.19)	33 (38.37)	15 (17.44)	82.56

### Inflammatory markers between 2 groups

CRP levels were 5.48±2.33 mg/L in the observation group and 6.12±2.79 mg/L in the control group before nursing. After nursing, they decreased to 2.03±1.19 mg/L and 3.76±1.45 mg/L, respectively, with no significant difference (P=0.097). ESR levels significantly reduced in the observation group from 22.4±5.62 mm/hr to 12.7±4.12 mm/hr, compared to the control group (24.5±6.31 mm/hr to 18.3±5.08 mm/hr, P<0.05). IL-6 levels significantly decreased in the observation group from 19.4±8.21 pg/mL to 8.24±3.42 pg/mL, compared to the control group (20.9±9.56 pg/mL to 13.57±4.92 pg/mL, P<0.01). IL-10 levels increased in both groups but with no significant difference (P=0.075). TNF-α levels significantly decreased in the observation group from 45.7±14.3 pg/mL to 24.5±8.72 pg/mL, compared to the control group (48.3±16.5 pg/mL to 38.1±12.9 pg/mL, P<0.05) (Table 3).

**Table 3:** Comparison of inflammatory markers between groups before and after nursing.

Marker	Group	Before<br>Nursing	After<br>Nursing	P-value
CRP	Observation<br>group	5.48±2.33<br>mg/L	2.03±1.19<br>mg/L	0.097
	Control<br>group	6.12±2.79<br>mg/L	3.76±1.45<br>mg/L	
ESR	Observation<br>group	22.4±5.62<br>mm/hr	12.7±4.12<br>mm/hr	<0.05
	Control<br>group	24.5±6.31<br>mm/hr	18.3±5.08<br>mm/hr	
IL-6	Observation<br>group	19.4±8.21<br>pg/mL	8.24±3.42<br>pg/mL	<0.01
	Control<br>group	20.9±9.56<br>pg/mL	13.57±4.92<br>pg/mL	
IL-10	Observation<br>group	15.3±4.21<br>pg/mL	30.1±7.36<br>pg/mL	0.075
	Control<br>group	14.7±4.06<br>pg/mL	20.4±6.21<br>pg/mL	
TNF-α	Observation<br>group	45.7±14.3<br>pg/mL	24.5±8.72<br>pg/mL	<0.05
	Control<br>group	48.3±16.5<br>pg/mL	38.1±12.9<br>pg/mL	

### Induration diameter evaluation

The results exhibited no significant difference in induration diameter between 2 groups before nursing (P>0.05). After nursing, the induration diameter of parturients in the observation group was 8.11±3.12 mm, presented higher relative to that of 1.30±0.31 mm in the control group (P<0.05). Table 4

**Table 4:** Induration diameter evaluation in both groups.

Groups	N	Observation<br>group	Control<br>group	t	P
Before<br>nursing	86	11.69±3.94	12.04±3.22	1.127	>0.05
After<br>nursing	86	8.11±3.12	1.30±0.31	5.731	<0.001
t		3.715	6.733		
P		0.036	<0.001		

## Discussion

Our study demonstrates that manual lymphatic drainage (MLD) and abdominal breathing significantly improve postpartum recovery by reducing perineal oedema, alleviating pain, and enhancing maternal comfort. This intervention led to quicker recovery times, lower pain scores, and higher comfort levels than routine midwifery care. The observation group also showed a notable reduction in inflammatory markers such as IL-6 and TNF-α, suggesting a beneficial effect on the inflammatory process. These results align with other studies highlighting the importance of physical interventions in postpartum rehabilitation.

Perineal lymphatic drainage is crucial in improving postpartum perineal oedema and pain and promoting postpartum rehabilitation. Local oedema and pain are important factors affecting postpartum activities, breastfeeding and rehabilitation, which can lead to urinary retention and constipation [13] [14]. In addition, oedema may affect perineal blood circulation, delay wound healing, and cause discomfort and pain. Therefore, finding a way to relieve maternal perineal swelling and pain is urgent.

Damage to the lymphatic system after surgery or for other reasons may cause lymph fluid to accumulate in the subcutaneous tissue’s superficial lymphatic vessels and interstitial fluid, resulting in local oedema. MLD opens up lymphatic pathways, soothes scar tissue, promotes lymphatic fluid reflux, and reduces local oedema by stroking superficial lymph. At present, this method has been applied to the treatment of upper limb oedema, lower limb lymphoedema after breast cancer surgery, and vulvar oedema after cervical cancer surgery. It has achieved good efficacy [15] [16]. According to the lymphatic distribution of the female perineum, the lymphatic vessels of the clitoris move forward into the pubis, and the lymphatic vessels of the labia minora move forward in line with those of the clitoris and move laterally into the lymphatic capillary vessels of the labia majora. The lymphatic vessels synthesised by the capillary lymphatic network of the labia majora basically go up along the labia majora and generally do not exceed the scope of the outer margin of the labia majora. However, after reaching the monsveneris, part of the lymphatic vessels can be injected into the contralateral inguinal lymph nodes through the monsveneris. Prolonged perineal pressure, impaired blood circulation, and perineal tissue damage during childbirth can cause perineal lymphatic reflux to be blocked, leading to perineal oedema. Through perineal MLD, the superficial lymphatic vessels and tissue fluid of the vulva tissue can be gradually drained to the abdomen, reducing the obstruction and exudation of the lymphatic vessels of the vulva, promoting the return of lymphatic fluid and effectively relieving oedema.

Abdominal breathing is great for calming emotions. When people are anxious and impatient, there will be shortness of breath. Deep and slow breathing movements can stimulate the parasympathetic nerve, reduce the level of sympathetic nerve activity, restore the balance of the autonomic nerve, stabilise people’s emotions quickly, and reduce the physical and mental symptoms of irritability, anxiety and tension. Therefore, pregnant women can do deep abdominal breathing at the beginning of contractions to help stabilise emotions and relieve contraction pain [17].

In this study, abdominal breathing combined with MLD technology was used for delivery, and the treatment initiation time, perineal oedema regression time and healing time in the observation group presented relatively shorter relative to the control group, which proved that this delivery mode was safe and reliable. Meanwhile, the degree of pain in the observation group presented lighter, and the comfort level in the observation group presented better than the control group, indicating that the delivery program could effectively relieve the pain and promote the comfort level. In addition, the observation group’s SAS score and SDS score significantly declined, and the clinical efficacy of the observation group presented substantially better relative to the control group, indicating that the treatment program had good clinical efficacy. The reason may be that the core content of the abdominal breathing method is to correctly guide the women in different stages of labour to take the corresponding breathing method, the pain and muscle tension during the contraction into active muscle relaxation, to lessen the pain caused by the contraction and the birth canal extrusion during the labour process. In addition, MLD can inhibit sympathetic nerve activity and enhance parasympathetic nerve activity to achieve analgesic effect. Cardiac massage can stimulate superficial lymphatic vessels, making lymphatic fluid flow from distal to proximal and significantly improve lymphatic systolic function.

In addition to its effects on pain and oedema, the combined use of abdominal breathing and MLD may also influence inflammatory responses, critical in postpartum recovery. The significant reductions in IL-6 and TNF-α levels observed in the observation group suggest that this treatment approach may help modulate the inflammatory process [18]. IL-6, a key mediator in inflammation, is known to increase in response to tissue injury, while TNF-α regulates immune cells and inflammation. By reducing these markers, abdominal breathing combined with MLD may alleviate physical symptoms like pain and oedema and promote a more favourable immune response during the postpartum period.

While CRP and IL-10 showed no significant differences between the two groups, the overall trends suggest that the treatment may still have some modulating effect on the inflammatory markers. CRP is a widely recognised marker for systemic inflammation. Although no significant difference was noted, the observed reduction in the observation group may hint at potential benefits that could be seen with larger sample sizes or more extended follow-up periods. IL-10, an anti-inflammatory cytokine, did not show a significant change, possibly due to the complex nature of its regulation during the postpartum period.

Lozowchuk and colleagues examined how partner support impacts inflammatory markers like IL-6:IL-10 during pregnancy and postpartum, finding that lower support was linked to higher inflammatory responses [19]. Similarly, our study found that manual lymphatic drainage (MLD) and abdominal breathing reduced IL-6 and TNF-α levels in postpartum women with perineal oedema. This suggests that physical interventions can help modulate inflammation and promote recovery. Both studies highlight the importance of managing inflammation for better postpartum health, focusing on emotional support and physical rehabilitation.

Lewis and colleagues found that in women with pre-eclampsia, maternal TNF-α levels were strongly correlated with IL-10 levels but not with IL-6 or IL-8, suggesting distinct regulatory patterns between these cytokines in maternal circulation [20]. Similarly, our study demonstrated that postpartum interventions like manual lymphatic drainage (MLD) and abdominal breathing significantly reduced IL-6 and TNF-α levels in women with perineal oedema, indicating that inflammation regulation is key to recovery. Both studies underscore the importance of balancing proinflammatory and anti-inflammatory cytokines for optimal maternal health. However, they focus on different conditions – pre-eclampsia in Lewis et al.’s [21] work and postpartum recovery in ours.

These findings support the hypothesis that abdominal breathing combined with MLD can provide more than just symptomatic relief; it may also reduce inflammation and enhance the body’s natural recovery processes after childbirth [22]. Future studies should further explore the relationship between these inflammatory markers and the efficacy of this combined treatment.

This study has several limitations, including a small sample size and a short follow-up period, which may affect the generalizability of the results. The potential influence of confounding factors, such as variations in childbirth severity or other postpartum care, was not fully addressed. Additionally, the lack of significant changes in inflammatory markers like IL-10 and CRP suggests further investigation to understand their role in postpartum recovery better. Larger, more diverse studies are needed to confirm these findings and explore long-term effects.

In conclusion, this study supports the hypothesis that combining MLD with abdominal breathing alleviates perineal oedema and pain and modulates the inflammatory response, promoting faster and more effective postpartum recovery. Although the findings are promising, future research with larger sample sizes and longer follow-ups is needed to investigate this intervention’s long-term effects and broader applicability in postpartum care further.

## Dodatak

### Acknowledgements

We would like to thank all the participants who contributed to this study. We also thank the medical staff at Affiliated Hospital 2 of Nantong University, Nantong First People’s Hospital, for their support and assistance during data collection.

### Funding

This work was supported by the Nantong Health Commission Research Project (Grant Numbers: MSZ2022016, MSZ2022020).

### Authors’ contributions

Lili Xue and Yaqiong Jiang contributed equally to this work, including study design, data collection, and manuscript drafting. Yongli Guo, Hongyan Ma, and Lingling Jiang participated in data collection and statistical analysis. Xiaoyu Wang, Xinyi Kang, and Ying Wang contributed to literature review and manuscript revision. Jiachen Cao and Liping Chen supervised the study and provided critical revisions to the final manuscript.

### Data availability statement

The data used in this study are available from the corresponding author upon reasonable request.

### Ethical approval

This study was conducted following the ethical guidelines of the Affiliated Hospital 2 of Nantong University, Nantong First People’s Hospital and approved by the hospital’s ethics committee. Informed consent was obtained from all participants before their inclusion in the study.

### Conflict of interest statement

All the authors declare that they have no conflict of interest in this work.
